# Clinical development methodology for infusion-related reactions with monoclonal antibodies

**DOI:** 10.1038/cti.2015.14

**Published:** 2015-07-17

**Authors:** Lucette Doessegger, Maria Longauer Banholzer

**Affiliations:** 1 Safety Risk Management, Licensing and Early Development (LEAD), F. Hoffmann-La Roche AG, Basel, Switzerland

## Abstract

Infusion-related reactions (IRRs) are common with monoclonal antibodies (mAbs) and timely related to drug administration and have been reported as anaphylaxis, anaphylactoid reactions and cytokine release syndrome, among other terms used. We address risk management measures for individual patients and for the study and propose a consistent reporting approach in an attempt to allow cross-molecule comparisons. Once the symptoms of IRR have resolved, the mAb may be restarted. Rechallenge should not be done for suspected IgE-mediated anaphylaxis and Grade 4 IRRs. Management of IRRs for subsequent patients includes administration of premedication, which, however, does not prevent IgE-mediated anaphylaxis. Reporting approach: (1) Report as IRRs, reactions occurring during or within 24 h after an infusion. Negative skin Prick test and absent or undetectable allergen-specific IgE levels have high negative predictive value for an IgE-mediated allergic reaction. If IgE-mediated anaphylaxis is suspected based on medical history and/or laboratory test results, the reaction should be reported as suspected (IgE mediated) anaphylaxis. (2) Collect signs and symptoms with grades to allow characterization of IRRs. IRRs pathogenesis is of scientific interest and has impact on drug development. Animal toxicology studies are neither predictive of severe IRRs nor of anaphylaxis in human. Preclinical tests should be further developed to identify patients at risk for severe IRRs, for complement activation-related pseudoallergy and for IgE-mediated anaphylaxis. The proposed approach should help standardizing data collection and analysis of IRRs in an attempt to enable comparisons across molecules.

Infusion-related reactions (IRRs) raise several issues with regard to diagnosis, evolution, reporting and risk management activities to offer patient access to specific therapies following clarification of the event.

IRRs are common adverse drug reactions (ADRs) with monoclonal antibodies. Symptoms are timely related to the drug administration and may range from symptomatic discomfort to fatal events. IRR as defined by Kang and Saif^1^ are ‘any signs or symptoms experienced by patients during the infusion of pharmacologic or biologic agents or any event occurring on the first day of drug administration'. IRRs have been reported, among other terms used, as anaphylaxis, anaphylactoid reactions and, cytokine release syndrome. Combinations of IRR types may occur in the same patient. Tumor lysis syndrome (TLS) has a specific clinical presentation, may occur spontaneously and is considered a separate entity. Symptoms of the reported terms may overlap which makes it difficult to establish a definite diagnosis without further investigations.

## Methods

We summarize the definitions of terms used to report IRRs, outline the problem with the reporting of reactions associated with the administration of mAbs and the regulatory position from FDA and EMA.

## Definitions of terms used to report IRRs

### Anaphylaxis

Applying the Coombs and Gell Classification,^2^ anaphylaxis is an example of a type I hypersensitivity reaction. However, the definition of anaphylaxis in the literature is evolving and suggests that there is no consensus with regards to the pathogenesis of anaphylaxis, in particular, whether non-IgE mechanisms may be involved as illustrated by the examples below.

1. Joint Task Force on Practice Parameters for drug allergy, representing the American Academy of Allergy, Asthma and Immunology (AAAAI), the American College of Allergy, Asthma and Immunology (ACAAI) and the Joint Council of Allergy, Asthma and Immunology (JCAAI):
   ‘Anaphylaxis is an immediate systemic reaction that occurs when a previously sensitized individual is re-exposed to an allergen. It is caused by rapid IgE-mediated immune release of vasoactive mediators from tissue mast cells and peripheral basophils with a potential late component.'^3^
2. World Allergy Organization (WAO):
   ‘Anaphylaxis is a severe, life-threatening generalized or systemic hypersensitivity reaction. The term allergic anaphylaxis should be used when the reaction is mediated by an immunologic mechanism, for example, IgE, IgG and immune complex complement related. An anaphylactic reaction mediated by IgE antibodies, such as peanut-induced food anaphylaxis, may be referred to as IgE-mediated allergic anaphylaxis. Anaphylaxis from whatever non-immunologic cause should be referred to as nonallergic anaphylaxis.'^4^
3. National Institute of Allergy and Infectious Disease (NIAID) and Food Allergy and Anaphylaxis Network (FAAN):
   ‘Anaphylaxis is a serious allergic reaction that is rapid in onset and may cause death.'^5^
4. Joint Task Force on Practice Parameters for anaphylaxis, representing the AAAAI; the American College of Allergy, Asthma and Immunology (ACAAI); and the Joint Council of Allergy, Asthma and Immunology (JCAAI):
  ‘A condition caused by an IgE-mediated reaction'^6^ (2005 update of the anaphylaxis practice parameter). ‘Anaphylaxis is an acute, life-threatening systemic reaction with varied mechanisms, clinical presentations and severity that results from the sudden systemic release of mediators from mast cells and basophils'.^7^ (2010 update).
5. European Academy of Allergy and Clinical Immunology (EAACI):
   ‘Severe, life-threatening systemic hypersensitivity reaction. This is characterized by being rapid in onset with life-threatening airway, breathing, or circulatory problems and is usually, although not always, associated with skin and mucosal changes.'^8^

Anaphylaxis usually starts within seconds to minutes of antigen exposure although time to onset varies according to publications. The clinical diagnosis of anaphylaxis is based on the Sampson criteria^5^ which are shown in Table 1.

It was thought that at least 80% of anaphylactic reactions should be identified by criterion 1 because the majority of anaphylactic reactions include skin symptoms, even when the allergic status of the patient and potential cause of the reaction may be unknown. However, cutaneous symptoms may be absent (in up to 20% of anaphylactic reactions, for example, in children with food allergy or insect-sting allergy). Consequently, in patients with a known allergic history and possible exposure, criterion 2 was believed to provide ample evidence that an anaphylactic reaction was occurring. Gastrointestinal symptoms were included as a pertinent target response because they have been associated with severe outcomes in various anaphylactic reactions. Criterion 3 was thought to identify the rare patients who experience an acute hypotensive episode after exposure to a known allergen.^5^

After 1st exposure, naive B cells produce IgM against the allergen.^9^ In the primary response to a thymus-dependent antigen, there is a logarithmic increase in serum IgM antibodies from 4 to 10 days.^10^ The half-life of IgM is about 5 days.^11^ This increase reflects clonal expansion and antibody production. It takes weeks to produce memory B cells and high-affinity plasma cells. After re-exposure, memory B cells are activated. Antibody production switches from IgM to IgE either via IgG with somatic hyper mutations, the most common path with high dose of antigen, or directly to IgE with much less mutations, the most common path with low antigen dose.^12^ Production of IgG takes about 3 weeks. The half-life of circulating IgE is 2 days, however, IgE are also membrane bound to mast cells, basophiles and may remain for weeks.^13^

### Anaphylactoid reaction

Anaphylactoid reactions or pseudoallergic reactions are immediate systemic reactions that mimic anaphylaxis but are caused by non-IgE-mediated release of mediators from mast cells and basophils.^3^ Anaphylactoid reactions may occur with the first exposure to an antigen and may be clinically indistinguishable from anaphylaxis. Unlike anaphylactic reactions, anaphylactoid reactions are milder upon repeated administration. However, there does not appear to be consensus as to whether anaphylactoid reaction is a separate entity from anaphylaxis.

The World Allergy Organization (WAO) has proposed that reactions without an immunologic mechanism should be referred to as nonallergic anaphylaxis, and the term anaphylactoid not to be used any longer.^14^

During the second National Institute of Allergy and Infectious Disease/Food Allergy and Anaphylaxis Network Symposium in 2006, the need was identified to further investigate the pathophysiological mechanisms and appropriate treatment of anaphylactoid or pseudoallergic reactions, fulfilling the diagnostic criteria for anaphylaxis with no involvement of an IgE-mediated mechanism.^5^

In 2010, the Joint Task Force on Practice Parameters, representing the AAAAI; the American College of Allergy, Asthma and Immunology (ACAAI); and the Joint Council of Allergy, Asthma and Immunology, the term anaphylaxis was continued to be used for IgE-mediated reactions for the purpose of the Practice Parameter document, while non-IgE-mediated reactions producing the same clinical response were referred to as anaphylactoid although it was acknowledged that the World Allergy Organization (WAO) had suggested that the term ‘anaphylactoid reaction' be eliminated.^7^

### Complement activation-related pseudoallergy

Complement activation-related pseudoallergy (CARPA) has been reported as a hypersensitivity reaction, the symptoms of which fit into the Coombs and Gell's Type I category, that is not initiated or mediated by pre-existing IgE antibodies but arises as a consequence of activation of the complement system.^15^ Complement activation leads to the liberation of C3a, C5a and C5b-9, which triggers mast cells, basophils and other phagocytic cells, via their specific receptors, for the secretion of a score of vasoactive mediators. The hemolytic complement assay (CH50) measures complement consumption.^16, 17^

Drugs causing CARPA include liposomal drugs, for example, liposomal doxorubicin (Doxil, ALZA Corporation, Bedford, OH, USA) and Caelyx (Janssen Pharmaceutica NV, Beerse, Belgium), radiocontrast media, micelle-solubilized drugs, for example, Taxol (paclitaxel, Hospira UK Limited, Maidenhead, UK) and Taxotere (docetaxel, Sanofi-Aventis U.S. LLC, Bridgewater, NJ, USA), antibodies (for example, rituximab and infliximab) among other drugs.^18^

CARPA typically occurs within minutes after starting the infusion. However, it may be delayed, particularly in premedicated patients. Almost all organs can be affected, the most frequent symptoms being flushing, rash, dyspnea, chest pain, back pain and subjective distress. Features distinguishing CARPA from the classical IgE-mediated reactions include:

1. Reaction arises mostly at first treatment, with no prior exposure. Rarely CARPA can occur at the second or third treatment. This may be explained by a greater amount of premedications and slow drug administration in the initial cycle
  - Reaction is milder or absent upon re-exposure
  - Spontaneous resolution of the reaction
  - Reaction may be tachyphylactic
  - Response to infusion speed
  - Response to steroid and antihistamine premedication
  - High reaction rate (2–10% or higher)
  - Reaction is unpredictable by standard allergy tests

Methods for preventing or reducing CARPA include the use of a variety of premedications with antihistamines and corticosteroids, and/or a reduction of the administration rate of pseudoallergenic drugs.^12, 18^

The severe form of CARPA is a type of anaphylactoid reaction.^15^

### Cytokine release syndrome

Cytokine release syndrome (CRS) is a symptom complex, which was first described with OKT3,^19, 20^ most recently with TGN1412^21^ but also with other monoclonal antibodies such as rituximab.^22^

CRS is believed to be a result of the sustained activation of a variety of cell types such as monocytes and macrophages, T cells and B cells, and is characterized by an increase in levels of TNFα and IFNγ within 1 to 2 h of stimulus exposure, followed by increases in interleukin (IL)-6 and IL-10 and, in some cases, IL-2 and IL-8. The principal mechanisms by which mAb-mediated CRS develops include signaling on target or non-target cells.^23^

A recent paper by Lee *et al.* suggests that IL-6 is a central mediator of toxicity in CRS. Therefore, tocilizumab, a humanized monoclonal antibody against the IL-6 receptor (IL-6R), has been proposed as a treatment, based on uncontrolled studies demonstrating that immunosuppression using tocilizumab with or without corticosteroids, can reverse CRS. However, because early immunosuppression could limit the efficacy of immunotherapy, the current approach aims at limiting tocilizumab to severe and life-threatening CRS.^24^

Premedication with corticosteroids has been reported to be effective in reducing the severity of symptoms caused by cytokine release, in addition to other management measures such as infusion rate reduction and fractionated dosing.^25^

## Outlining the problem

### Diagnosis of anaphylaxis based on the clinical presentation—Sampson criteria

The Sampson criteria^5^ were intended to be used by both the medical and lay community to manage emergencies at school and in ER and are applicable for immediate therapeutic measures when facing clinical symptoms consistent with anaphylaxis. Monoclonal antibodies may be associated with infusion-related reactions meeting the Sampson criteria for anaphylaxis. In such instances, the diagnosis of allergic anaphylaxis should be further investigated as this has impact on rechallenge of the patient.

### Evolution of National Cancer Institute Common Terminology Criteria For Adverse Events: categories and grading

For studies performed in oncology, the National Cancer Institute Common Terminology Criteria for Adverse Events (NCI CTCAE) is used for the grading of adverse events.

In NCI CTCAE version 3,^26^ anaphylaxis was by default of grade 4 under the category Allergy/Hypersensitivity. There was in addition a category for cytokine release syndrome/acute infusion reaction with the following remark for this category: ‘Cytokine release syndromes/acute infusion reactions are different from Allergic/hypersensitive reactions, although some of the manifestations are common to both AEs. An acute infusion reaction may occur with an agent that causes cytokine release (for example, monoclonal antibodies or other biological agents). Signs and symptoms usually develop during or shortly after drug infusion and generally resolve completely within 24 hrs of completion of infusion. Signs/symptoms may include: Allergic reaction/hypersensitivity (including drug fever); Arthralgia (joint pain); Bronchospasm; Cough; Dizziness; Dyspnea (shortness of breath); Fatigue (asthenia, lethargy, malaise); Headache; Hypertension; Hypotension; Myalgia (muscle pain); Nausea; Pruritis/itching; Rash/desquamation; Rigors/chills; Sweating (diaphoresis); Tachycardia; Tumor pain (onset or exacerbation of tumor pain due to treatment); Urticaria (hives, welts, wheals); Vomiting.'

In NCI CTCAE version 4,^27^ allergic reaction and anaphylaxis are two distinct categories and the anaphylaxis grading starts with grade 3 (no grade 1 and 2) and goes up to grade 5 (death). An allergic reaction is defined as a disorder characterized by an adverse local or general response from exposure to an allergen while anaphylaxis is defined as a disorder characterized by an acute inflammatory reaction resulting from the release of histamine and histamine-like substances from mast cells, causing a hypersensitivity immune response, clinically presenting with breathing difficulty, dizziness, hypotension, cyanosis and loss of consciousness and potentially leading to death. Thus, the definition for anaphylaxis in version 4 of NCI CTCAE reflects that of the AAAAI/ACAAI/JCAAI 2010.^3^

NCI CTCAE version 4 newly includes separate categories for ‘infusion-related reaction' and ‘cytokine release syndrome'. Infusion-related reaction is defined as a disorder characterized by adverse reaction to the infusion of pharmacological or biological substances and CRS is defined as a disorder characterized by nausea, headache, tachycardia, hypotension, rash and shortness of breath and caused by the release of cytokines from the cells. The descriptions of the grades for the four categories, allergic reaction, anaphylaxis, infusion-related reaction and CRS, show a high degree of shared terminology (see Table 2).

## Under/over reporting

Owing to current reporting practices predominantly based on clinical presentation, investigators, health care professionals and pharmaceutical companies are using inconsistent terminology for reporting IRRs leading to under/over reporting of allergic reactions, in particular anaphylaxis. Furthermore, the use of premedication and concomitant treatment may impact on the occurrence as well as the spectrum of hypersensitivity reactions associated with the administration of therapeutic biologics. Therefore, no comparison should be made regarding incidence of IRRs or hypersensitivity reactions across drugs.

## Cross reactivity

Occurrence of a reaction with the first infusion of a mAb is unlikely to be an allergic, that is, IgE-mediated reaction—although cross-reactivity may be the cause of an allergic reaction including anaphylaxis.

Cetuximab, a chimeric mouse–human IgG1 monoclonal antibody against the epidermal growth factor receptor (EGFR), approved for use in colorectal cancer and squamous cell carcinoma of the head and neck, has been reported to cause severe hypersensitivity reactions, initially with low incidence (1–3%) and later at levels reaching 22% depending on the geographic location.^28^ After analysis of the pretreatment serum samples of a few subjects, results have found IgE against cetuximab in the majority of patients who experienced hypersensitivity reactions to the drug as defined by a prespecified ‘hypersensitivity' case definition graded as per NCI CTCAE version 3 for Allergic reaction/hypersensitivity (including drug fever) and based on the presence or absence of a reaction within 2 h after the administration of cetuximab. These IgE antibodies were shown to be specific for an oligosaccharide, galactose-α-1,3-galactose, which is present on the Fab portion of the cetuximab heavy chain.^29^ The presence of such IgE antibodies before treatment may put patients who receive monoclonal antibodies containing galactose-α-1,3-galactose at risk for hypersensitivity reactions.^29^ A blood test for IgE specific for the oligosaccharide on cetuximab would be the easiest and most reliable screening test to prevent these hypersensitivity reactions.^30^

## Current regulatory guidelines

There is no clarity from regulators with regards to reporting IRRs and anaphylaxis terms. Below are excerpts from EMA and FDA guidance.

### EMEA/CHMP/BMWP/14327/2006

GUIDELINE ON IMMUNOGENICITY ASSESSMENT OF BIOTECHNOLOGY-DERIVED THERAPEUTIC PROTEINS. December 2007.^31^

#### Consequences on Safety

##### Acute consequences

Usually, patients who develop antibodies are more likely to show infusion-related reactions. Acute infusion reactions including anaphylactic reactions may develop during (within seconds) or within a few hours following infusion. Applicants should differentiate between the terms ‘infusion reaction' and ‘anaphylaxis' and carefully define which symptoms to label as ‘infusion-related reaction'. ‘Infusion reactions' usually represent symptoms occurring in a close timely relationship to an infusion and are not necessarily linked to anaphylaxis or even hypersensitivity. However, acute reactions can be true allergic, namely IgE-mediated type I reactions (anaphylactic reactions), including hypotension, bronchospasm, laryngeal or pharyngeal edema, wheezing and/or urticaria. The term ‘anaphylaxis' should be restricted to such situations and represents a strict contraindication to further exposure to the drug. However, the majority of infusion reactions are characterized by more non-specific symptoms, for some products more frequently occurring on initial exposure and sometimes less frequent/severe reactions are observed on re-exposure. An infusion reaction might not represent a contraindication to further exposure. A range of symptoms including headache, nausea, fever or chills, dizziness, flush, pruritus, and chest or back pain have been described in relation to infusions. It is acknowledged that the distinction between an infusion reaction and anaphylaxis can be challenging, but nevertheless such distinction is necessary due to the different clinical consequence. Applicants should not only focus on infusion reactions and anaphylactic symptoms since the consequence of immunogenicity is product-specific and can elicit unexpected clinical symptoms.

### FDA DRAFT GUIDANCE FOR INDUSTRY

IMMUNOGENICITY ASSESSMENT FOR THERAPEUTIC PROTEIN PRODUCTS, August 2014.^32^

#### Consequences for safety

The safety consequences of immunogenicity may vary widely and are often unpredictable in patients administered therapeutic protein products. Therefore, a high index of suspicion should be maintained for clinical events that may originate from such responses, even if the initial risk assessment suggests a lower risk of immunogenicity. The applicant should provide a rationale for the proposed immunogenicity testing paradigm, based on product- and patient-specific concerns. The following sections describe a few of the major safety concerns associated with immunogenicity:

##### Anaphylaxis

Anaphylaxis is a serious, acute allergic reaction characterized by certain clinical features. The definition currently accepted by the Agency relies on clinical diagnostic criteria and does not specify a particular immunologic mechanism.^5^ Historically, the definition of anaphylaxis has invoked the involvement of specific IgE antibodies. However, such a mechanistic definition may be problematic in the context of therapeutic protein product development and other clinical settings where it may not always be possible to identify a specific immunologic mechanism as the basis of an adverse event. To capture all potential adverse events of interest, the Agency recommends identifying all cases meeting the clinical diagnostic criteria of anaphylaxis, regardless of the presumed pathophysiology. Additional information, such as the assessment of serum histamine, serum tryptase and complement components, following a reaction or the detection of product-specific IgE antibodies may help elucidate the pathophysiology of the anaphylactic response and thus guide control and mitigation strategies.

Furthermore, the presence of antidrug antibody (ADA) alone is not necessarily predictive of anaphylaxis or other hypersensitivity reactions. Correlation with clinical responses is typically required to determine the clinical relevance of these antibodies. Determination of the underlying mechanism remains of interest, however, because anaphylaxis with confirmation of IgE involvement has certain prognostic implications for repeat exposure, as well as for potential therapeutic options for mitigation.

##### Cytokine release syndrome

Cytokine release syndrome is a symptom complex caused by the rapid release of proinflammatory cytokines from target immune cells.^33, 34^ Although cytokine release syndrome is not directly related to immunogenicity, the clinical presentation of cytokine release syndrome overlaps with anaphylaxis and other immunologically related adverse reactions. Distinguishing this symptom complex from these other types of adverse reactions is potentially useful for the purpose of risk mitigation. Although the underlying mechanisms may not be fully understood, in some cases the mechanism appears to relate to the cross-linking of activating cell surface expressed receptors, which are the targets of the therapeutic protein product (for example, CD28 expressed on T cells). A risk-based evaluation, focused on the mechanism of action of the therapeutic protein product as well as results of animal and *in vitro* evaluations should be performed to determine the need for collection of pre- and post-dose cytokine levels in the early phase of clinical development. In case of a clinical adverse event, such an evaluation may provide evidence to support the clinical diagnosis of cytokine release syndrome and help distinguish this entity from other acute drug reactions (for example, anaphylaxis).

##### ‘Infusion Reactions'

Therapeutic protein products may elicit a range of acute effects, from symptomatic discomfort to sudden, fatal reactions that have often been grouped as ‘infusion reactions' in the past. Although the term implies a certain temporal relationship, infusion reactions are otherwise not well defined and may encompass a wide range of clinical events, including anaphylaxis and other event that may not be directly related to antibody responses, such as cytokine release syndrome. In the absence of an agreed-upon definition for infusion reaction, the categorization of certain adverse events as infusion reactions without further detail is problematic and is not recommended. Sponsors are encouraged to use more descriptive terminology when possible, noting the timing, duration and specific signs and symptoms observed upon administration of a therapeutic protein product and to provide data from mechanistic studies which may facilitate a mitigation strategy.

## Basis for reporting incidence of IRR

As illustrated by some examples included in Table 3, the analysis for reporting IRR incidences is based on heterogeneous definitions of IRRs with regards to selected terms, time window and additional parameters, for example, reversibility criteria. This information was taken from FDA approval packages for ofatuzumab, panituzumab, cetuximab, natalizumab and infliximab^35, 36, 37, 38, 39^ or the briefing document for belimumab.^40^ Thus, comparisons of IRRs incidences across molecules are not appropriate. Furthermore, caution should be exercised when comparing IRR incidences for therapeutic agents from the same class as underlying diseases and/or concomitant medications are also confounding factors.

## Discussion

The primary goal of characterization and reporting of IRRs with mAbs in clinical development is to identify reactions of allergic (IgE mediated) origin since this has implications on further rechallenge with the compound. Indeed, once a patient has experienced an IgE-mediated anaphylactic reaction to a therapeutic agent, the use of such an agent is contraindicated for that individual. The long-term strategy for the prevention of IgE-mediated anaphylaxis is the avoidance of the confirmed culprit drug. If avoidance is not possible and no alternative therapy is available, desensitization to the drug is indicated.^41^ Diagnosis of an IRR provides the option of re-challenging the patient with the therapy and of the use of precautionary measures to minimize subsequent IRRs.

In general, for reactions that occur with the first infusion of a mAb, no specific laboratory investigations are performed for the management of individual patients. However, special laboratory parameters, cytokines (IL2, IL6, IL8, IL10, TNF-α, INF-ϒ) and complement (for example, CH50, C3a, C5a (anaphylatoxins), SC5b-9), may be required in an early phase study to better understand the pathophysiology of the reaction and to enable a correlation between laboratory results and the clinical picture. Usually, reactions associated with CARPA or CRS respond to reduction of the infusion rate and premedication. Such reactions become less severe with subsequent infusions.^42^

It is important to note that Sampson criteria for anaphylaxis^5^ were developed to provide clinical criteria to the emergency responder or treating physician with a relatively simple and rapid means to make the diagnosis of anaphylaxis. Hence, the criteria are not meant to provide a definitive diagnosis but to guide emergency management of individuals.

If anaphylaxis of allergic origin is suspected with the administration of a mAb, for example, there is a rapid onset of symptoms, new occurrence of an IRR beyond the first infusion or an increase in severity of the reaction at subsequent infusions, the measurement of serial tryptase is recommended and a skin Prick test should be considered.

Tryptase is a rather specific mast cell derived mediator and a rapid increase in serum tryptase from baseline is considered a reliable marker for mast cell activation.^41^ In primary mast cell disease (mastocytosis) baseline levels of total tryptase are usually elevated.^43^ Basophils also contain and release tryptase, but they do not represent important contributors to tryptase levels. Basophils may release tryptase following IgE-mediated activation. Tryptase load is highly variable both in mast cells and in basophils. Serial serum tryptase determination should include the peak tryptase value (optimally measured between 30 min and 2 h after the onset of symptoms) and a baseline value (accurate baseline values are measured best 48–72 h after the anaphylaxis episode) allowing a comparison between the peak and the baseline values.^44^ During IgE-mediated anaphylaxis, allergen-induced cross-linking of IgE-binding sites on mast cells is followed by an explosive release of granular mediators.^41^

However, Sala-Cunill *et al.* showed that tryptase is not an optimal biomarker for the diagnosis of anaphylaxis as clinically defined by the National Institute of Allergy and Infectious Disease (NIAID) and Food Allergy and Anaphylaxis Network.^5^ Tryptase levels were shown to be normal in 36.6% of patients during the acute episode of anaphylaxis^44^ diagnosed clinically.^5^

It is important to note that also CARPA is associated with an increase in tryptase. However, the clinical picture, that is, CARPA develops within minutes after starting the first infusion, helps differentiating CARPA from anaphylaxis of allergic origin.^17^

Thus, there are no laboratory tests for confirming the diagnosis of an IgE-mediated anaphylaxis at the time of presentation. Nevertheless, consider measuring drug-specific IgE levels in serum for the assessment of sensitization to relevant allergens ascertained from the history of the anaphylactic episode.^45^ Although IgE-mediated anaphylaxis occurs after re-exposure to an allergen, cross-reactivity to a component of the drug may occur after the first exposure in isolated cases, for example, galactose allergy and cetuximab.

## Risk management measures

Based on internal experience regarding treatment and prophylaxis of IRRs, the following risk management measures may be considered to decrease the occurrence and severity of IRRs:

### Related to individual patients

IRRs predominantly occur after first exposure to a drug. Patients experiencing IRRs should be treated symptomatically as required. Infusion interruption or infusion rate decrease should be considered based on the severity of symptoms.

### Related to drug development

The occurrence of moderate to severe IRRs with a specific mAb may be managed for future patients by introducing premedications (for example, paracetamol, NSAIDs, antihistamines and corticosteroids or a combination of these), decreasing the infusion rate or fractionating the dose.

In general, if severe IRRs are observed during dose escalation, standard premedication would be recommended for further patients and infusion rate reduction considered to manage the occurrence and severity of IRRs. Such measures are not indicated and not effective for IgE-mediated anaphylactic reactions.^42^

Following the very serious adverse reactions that occurred in the first-in-man clinical trial of TGN412 in March 2006,^21^ the ‘Minimal Anticipated Biological Effect Level' (MABEL approach) was implemented for ‘first-in-man' clinical trials. The MABEL is the anticipated dose level leading to a minimal biological effect level in humans.^46, 47^

## Prediction of IRRs

### Test for cytokine release

Anaphylaxis and cytokine release are not predicted from animal toxicology studies. In order to improve the ability to predict cytokine release in support of first in-human studies, *in vitro* cytokine release assays in human whole blood are used. However, these assays are neither able to predict the severity of the reaction nor to identify patients at risk for severe IRRs.

Indeed, cytokine release assays should be considered as a hazard identification tool and not an accurate and reliable risk quantification tool.^48^

### Tests for CARPA

Various preclinical tests have been proposed to predict the risk of CARPA.^17^ As for the cytokine release assays in human whole blood, these tests are not helping in identifying patients at risk for severe CARPA.

## Proposed reporting approach

A consistent reporting approach in clinical trials using the term infusion-related reaction should enable comparisons of incidences of IRRs across molecules.

For reactions which are timely related to an infusion (that is, occurring during or within 24 h of completion of the infusion) IRR should be reported. This includes reactions fulfilling Sampson criterion 1.

The reported term should be revised to suspected anaphylaxis of allergic origin, if Sampson criterion 2 is fulfilled, that is, when there is suspicion of presensitization. Note: patients known to be allergic to a component of the drug product should not receive the drug.

When reactions are reported as IRRs, it is imperative to also collect signs and symptoms thereof, including their grades, in order to characterize the IRRs associated with a specific mAb.

**Figure 1:**
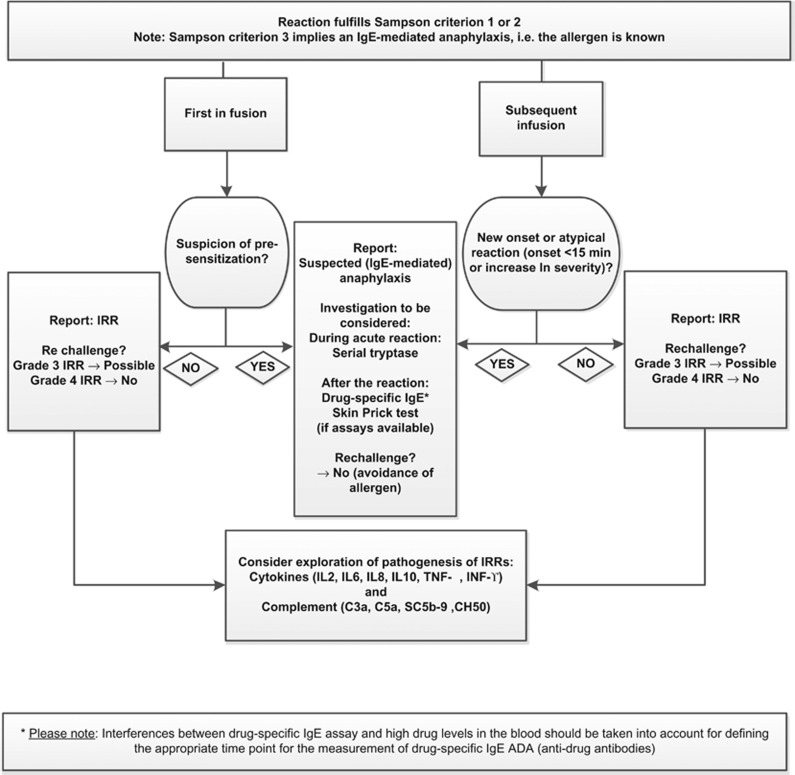
IRR Algorithm.

An algorithm recommending how to report IRRs fulfilling Sampson criterion 1 and suspected anaphylaxis of allergic origin (that is, fulfilling Sampson criterion 2) is provided in Figure 1.

## Conclusions

IRRs are commonly seen with mAbs. The pathogenesis of IRRs is of scientific interest and has impact on drug development. Animal toxicology studies are not predictive of IRR severity or IgE-mediated anaphylaxis in human. Further tests such as *in vitro* tests for cytokine release in human whole blood and preclinical tests for CARPA and other *in vitro* tests should be further developed to identify patients at risk for severe IRRs or CARPA and for IgE-mediated anaphylaxis.

The mAb may be restarted once symptoms of the IRR have resolved. No rechallenge should be undertaken for suspected (IgE-mediated) anaphylaxis and for Grade 4 IRRs. Management of IRRs includes the administration of premedication which, however, does not prevent occurrence of IgE-mediated anaphylaxis.^42^

The proposed approach for characterizing and reporting IRRs should help standardizing collection and analysis methods for reactions occurring with the first infusion in an attempt to enable comparisons across molecules.

Following the clinical diagnosis of a reaction meeting Sampson criteria for anaphylaxis, it is difficult to confirm whether the reaction is IgE-mediated due to lack of standardized assays for drug-specific IgEs and of skin Prick tests. However, if IgE-mediated anaphylaxis is suspected based for example on, medical history, it is recommended to report the reaction as such.

## Figures and Tables

**Table 1:** Clinical criteria for diagnosing anaphylaxis^a^

*Anaphylaxis is highly likely when any one of the following three criteria are fulfilled:*	
**1.**	Acute onset of an illness (minutes to several hours) with involvement of the skin, mucosal tissue or both (for example, generalized hives, pruritus or flushing, swollen lips–tongue–uvula) and at least one of the following
**a.**	Respiratory compromise (for example, dyspnea, wheeze-bronchospasm, stridor, reduced PEF, hypoxemia)
**b.**	Reduced BP or associated symptoms of end-organ dysfunction (for example, hypotonia (collapse), syncope, incontinence)
	
**2.**	Two or more of the following that occur rapidly after exposure to a likely allergen for that patient (minutes to several hours):
**a.**	Involvement of the skin-mucosal tissue (for example, generalized hives, itch-flush, swollen lips–tongue–uvula)
**b.**	Respiratory compromise (for example, dyspnea, wheeze-bronchospasm, stridor, reduced PEF, hypoxemia)
**c.**	Reduced BP or associated symptoms (for example, hypotonia (collapse), syncope, incontinence)
**d.**	Persistent gastrointestinal symptoms (for example, crampy abdominal pain, vomiting)
	
**3.**	Reduced BP after exposure to known allergen for that patient (minutes to several hours):
**a.**	Infants and children: low systolic BP (age specific) or >30% decrease in systolic BP∗
**b.**	Adults: systolic BP of <90 mm Hg or >30% decrease from that person's baseline

Abbreviations: BP, blood pressure; PEF, peak expiratory flow.

PEF × Low systolic blood pressure for children is defined as <70 mm Hg from 1 month to 1 year, less than (70 mm Hg+[2 × age]) from 1 to 10 years, and <90 mm Hg from 11 to 17 years., Peak expiratory flow.

a Sampson HA *et al.*^5^

**Table 2:** Grading according to the NCI Common Terminology Criteria for Adverse Events version 4.03 (June 2010)

*Adverse event*	*Grade 1*	*Grade 2*	*Grade 3*	*Grade 4*	*Grade 5*
Anaphylaxis	—	—	*Symptomatic bronchospasm, with or without urticaria; parenteral intervention indicated; allergy-related edema/angioedema; hypotension*	Life-threatening consequences; urgent intervention indicated	Death
Infusion-related reaction	Mild *transient* reaction; infusion interruption not indicated; intervention not indicated	Therapy or infusion interruption indicated but responds promptly to symptomatic treatment (for example, antihistamines, NSAIDs, narcotics, IV fluids); prophylactic medications indicated for ⩽24 h	Prolonged; recurrence of symptoms following initial improvement; hospitalization indicated for clinical sequelae	Life-threatening consequences; urgent intervention indicated	Death
Allergic reaction	*Transient flushing or rash, drug fever <38 °C;* intervention not indicated	*Intervention or infusion interruption indicated; responds promptly to symptomatic treatment (for example, antihistamines, NSAIDs, narcotics); prophylactic medications indicated for*⩽*24 h*	Prolonged; recurrence of symptoms following initial improvement; hospitalization indicated for clinical sequelae *(for example, renal impairment, pulmonary infiltrates)*	Life-threatening consequences; urgent intervention indicated	Death
Cytokine release syndrome	Mild reaction; infusion interruption not indicated; intervention not indicated	Therapy or infusion interruption indicated but responds promptly to symptomatic treatment (for example, antihistamines, NSAIDs, narcotics, IV fluids); prophylactic medications indicated for ⩽24 h	Prolonged; recurrence of symptoms following initial improvement; hospitalization indicated for clinical sequelae *(for example, renal impairment, pulmonary infiltrates)*	Life-threatening consequences; *pressor or ventilator support indicated*	Death

Abbreviation: NSAID, nonsteroidal anti-inflammatory drugs

Italic and underlined are highlighting differences/variations in descriptives across entities.

**Table 3:** Examples of IRR definitions used for analysis

*Compound*	*IRR definition for analysis purpose*	*IRR time window*
Belimumab^a^	164 preferred terms, plus All preferred terms indicative of a hypersensitivity reaction 9 specific preferred terms related to ‘hypersensitivity reaction'	Onset on the infusion day and resolution within 7 days For hypersensitivity reaction: onset on the day of infusion irrespective of the resolution date Nine specific hypersensitivity reaction PTs: no duration requirement
Ofatumumab^b^	To perform the analysis of AEs potentially related to infusion reactions, all AEs in the dataset that occurred on day 0 or 1 after an infusion of ofatmumab were initially identified for review. However, AEs in the following SOCs were removed due to the low likelihood that they are infusion reactions: Infections, neoplasms, blood and lymphatic disorders, investigations, metabolism and nutrition disorders and injury. The following additional PTs that were removed from the analysis (because it is unlikely that these signs/symptoms were related to an infusion): Palatal dysplasia, hemorrhoids, feces discolored, deep vein thrombosis, thrombophlebitis superficial, pallor, petechie, ecchymosis, actinic keratosis, skin lesion, hemoptysis, epistaxis, interstitial lung disease, pleural effusion, hematuria, pollakiuria, insomnia, depression, anxiety, tendonitis, extravasation, catheter related complication, rectal hemorrhage and stomatitis. The remaining 84 PTs in the dataset formed the basis of infusion reactions. This should be considered a conservative analysis of infusion reactions, recognizing that it is not possible to be completely accurate in the attribution of these AEs.	All AEs that occurred on day 0 or 1 after an infusion of ofatumumab
Panitumumab^c^	40 prespecified terms indicating any signs and symptoms of potential infusion reaction defined per CTCAE Version 3.0 as ‘allergic reaction/hypersensitivity' and ‘cytokine release syndrome/acute infusion reaction' and coincident with any panitumumab infusion, Terms: allergic reaction, fatigue, myalgia, anaphylaxis, fever, nausea, angioedema, flushing, pruritus, arthralgia, headache, rash, asthenia, hives, rigors, bronchospasm, hypersensitivity, sweating, chills, hypertension, tachycardia, cough, hypotension, tumor pain, desquamation, infusion reaction, urticaria, diaphoresis, itching, vomiting, dizziness, joint pain, welts, drug fever, lethargy, wheals, dyspnea/dyspnea malaise, muscle pain, edema/oedema.	Onset on the day of the infusion and resolution within 24 h
Cetuximab^d^	Any event described at any time during the clinical study as ‘allergic reaction' or ‘anaphylactoid reaction, plus Any event described as ‘allergic reaction', ‘anaphylactoid reaction', ‘fever', ‘chills', ‘chills and fever' or dyspnea.	Allergic reaction AE: onset at any time during clinical study Prespecified AEs: onset on the first day of dosing
Natalizumab^e^	All adverse events	Onset within 2 h after the initiation of the study drug infusion
Infliximab^f^	Any adverse event	Onset during the infusion or within 1–2 h after the infusion

a FDA Advisory Committee Briefing Document for belimumab, 2010.

b FDA Approval Package for Arzerra (ofatumumab), 2009.

c FDA Approval Package for Vectibix (panitumumab), 2006.

d FDA Approval Package for Erbutux (cetuximab), 2004.

e FDA Approval Package for Tysabri (natalimumab), 2004.

f FDA Approval Package for Remicade (infliximab), 1998.
